# Supplementary material for: Neural circuit for social authentication in song learning
Source: Nat Commun. 2022 Aug 16;13:4442. doi: 10.1038/s41467-022-32207-1 (PMC9381780; doi:10.1038/s41467-022-32207-1)
Supplement: Supplementary file 1 — Supplementary Information [file 41467_2022_32207_MOESM1_ESM.pdf]

Supplementary Information

Neural Circuit for Social Authentication in Song Learning

Jelena Katic[1], Yuichi Morohashi[1] and Yoko Yazaki-Sugiyama[1,2]*

[1]Neuronal Mechanism for Critical Period Unit, Okinawa Institute of Science and Technology Graduate University, [2]WPI-IRCN, The University of Tokyo

* Corresponding Author

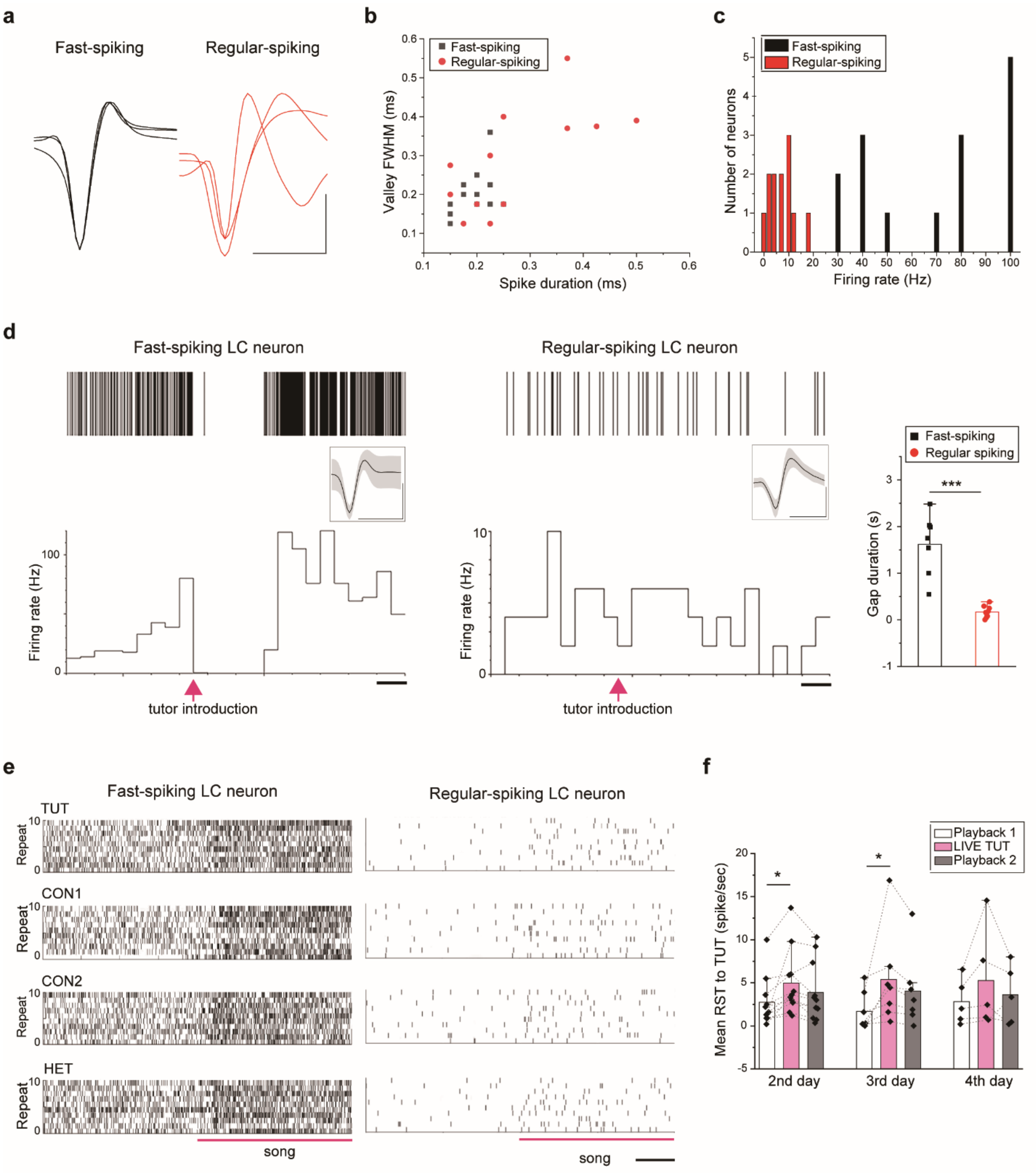

Supplementary Fig. 1: LC neurons increase auditory responses to song playback and fast-spiking LC neurons cease firing upon tutor introduction into the cage

a, Mean spike waveforms of fast-spiking and regular-spiking LC neurons (scale bars: 0.5 ms horizontal, 0.4 mV vertical). **b**, Scatter plots of spike duration against valley full width at half maximum values of fast-spiking and regular-spiking LC neurons. **c**, Cumulative histogram of LC neurons against their firing

rates (bin size: 2 Hz). Fast-spiking and regular-spiking neurons are separated at 20 Hz. **d** left and middle**,** Raster plots of spiking activity (top) and cumulative spike histogram (bottom) of single fast-spiking (**d** left) or regular-spiking (**d** middle) LC neuron responding to tutor introduction into the cage for the first time (pink arrow, scale bars: 0.5 s). Insets: spike waveform of LC neurons (mean ± s.d., scale bars: 0.5 ms horizontal, 0.4 mV vertical). **d** right, Gap duration in the spiking activity of fast- and regular-spiking LC neurons upon tutor introduction into the cage for the first time. **e,** Raster plots of spiking activities of single fast-spiking (**e** left) and regular-spiking (**e** right) LC neurons before and during playback of a different song (scale bars: 0.5 s). Time-alignment with the onset of a song playback (pink line indicates duration of the song playback). **f,** Mean response strength (RST) of LC neurons to tutor song playback (Playback 1 and 2) and tutor singing (LIVE TUT) throughout 2nd to 4th days of tutoring. N = 12 (**a-c**), N = 8 (**d**, **f:** 2nd day), N = 5 (**f:** 3rd and 4th day) n = 29 (**a-c**), n = 7 (**d:** fast-spiking), n = 9 (**d :** regular-spiking), n = 11 (**f:** 2nd day), n = 7 (**f:** 3rd day), n = 5 (**f:** 4th day). TUT: tutor song, CON1: conspecific song 1, CON2: conspecific song, HET: heterospecific song, N: number of birds, n: number of neurons. Mean ± s.e.m., *p = 0.049, 0.05, ***p < 0.001, Two-sided Mann-Whitney Rank Sum Test (**d**, **f**). Source data are provided as a Source Data file.

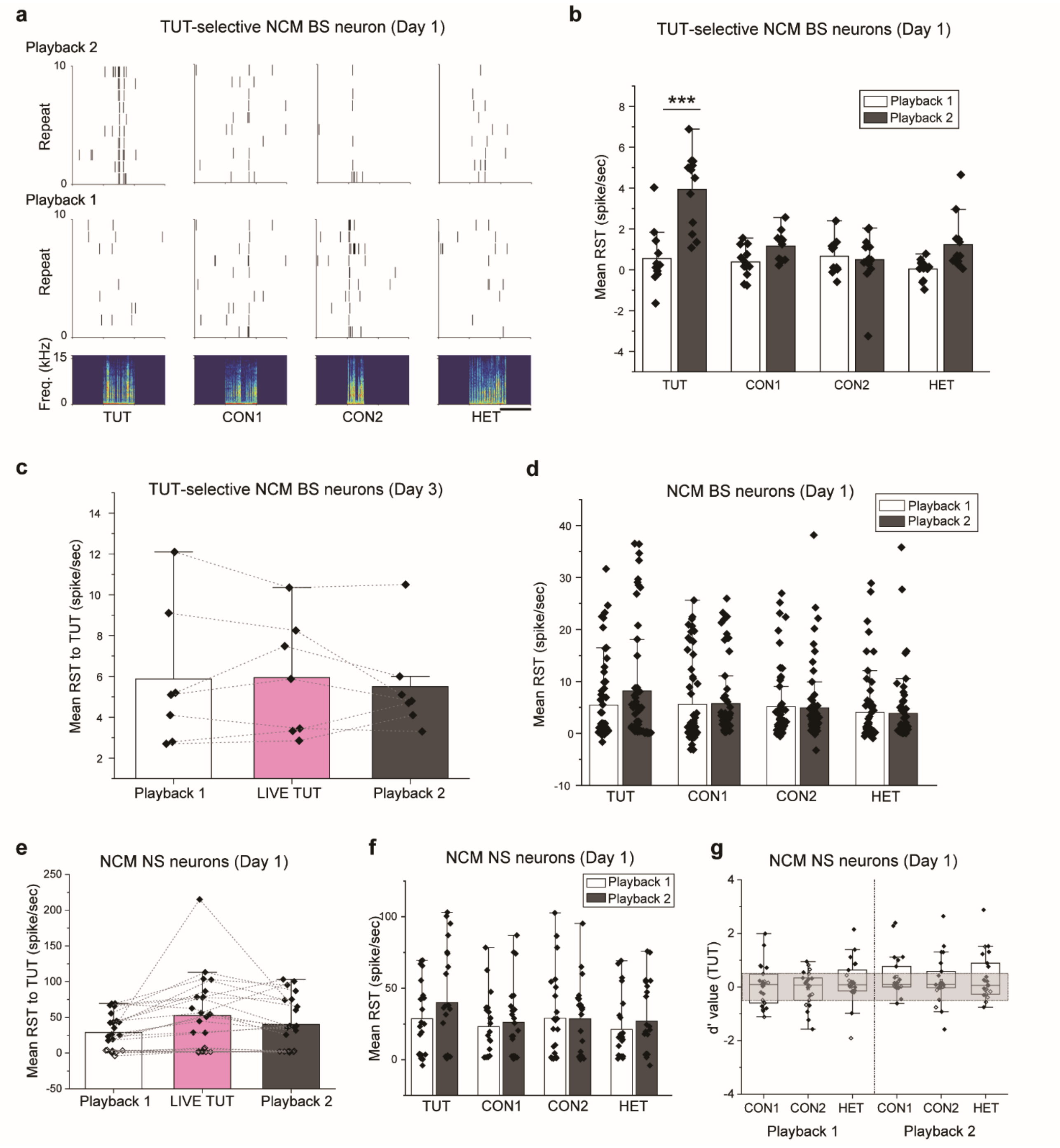

Supplementary Fig. 2; Broad-spiking NCM neurons develop selectivity to tutor song playback after two days of exposure to live tutor singing

a, Raster plots of spiking activity of a TUT selective BS NCM neuron to playback of different songs (song spectrograms shown in the bottom) before (Playback 1) and after (Playback 2) exposure to tutor singing (scale bars: 2 s). **b-f,** Mean response strength (RST) of TUT selective BS neurons (**b**, **c**) all BS NCM neurons (**d**), or all NS NCM neurons (**e**, **f**) to tutor song playback (Playback 1 and 2), LIVE TUT (**c**, **e**) or playback

of different songs before (Playback 1) and after (Playback 2) hearing the tutor singing (LIVE TUT) (**b**, **d**, **f**) during the first (**b**, **d**, **e**, **f**) or third (**c**) day of tutoring. **g**, Mean d-prime values for TUT over other song stimuli of all NS NCM neurons before (Playback 1) and after (Playback 2) hearing LIVE TUT. The boxes show the 25–75%, the center lines are defined by the median and open squares by the mean. The whiskers include all data points within 1.5 IQR (Interquartile range) and he 'outsider' dots are the data points that fall outside the whisker line. Grey areas indicate non-selective responses (-0.5<d' value<0.5). N = 5, n = 12 (**b**), n = 7 (**c**), n = 57 (**d**). n = 23 (**e-g**). BS: broad-spiking neuron, NS: narrow-spiking neuron, TUT: tutor song, CON1: conspecific song 1, CON2: conspecific song, HET: heterospecific song, N: number of birds, n: number of neurons. Mean ± s.e.m., ***p = 0.0000518, Two-sided Student T-test (**b**) or Two-sided Mann-Whitney Rank Sum Test (**c-f**). Source data are provided as a Source Data file.

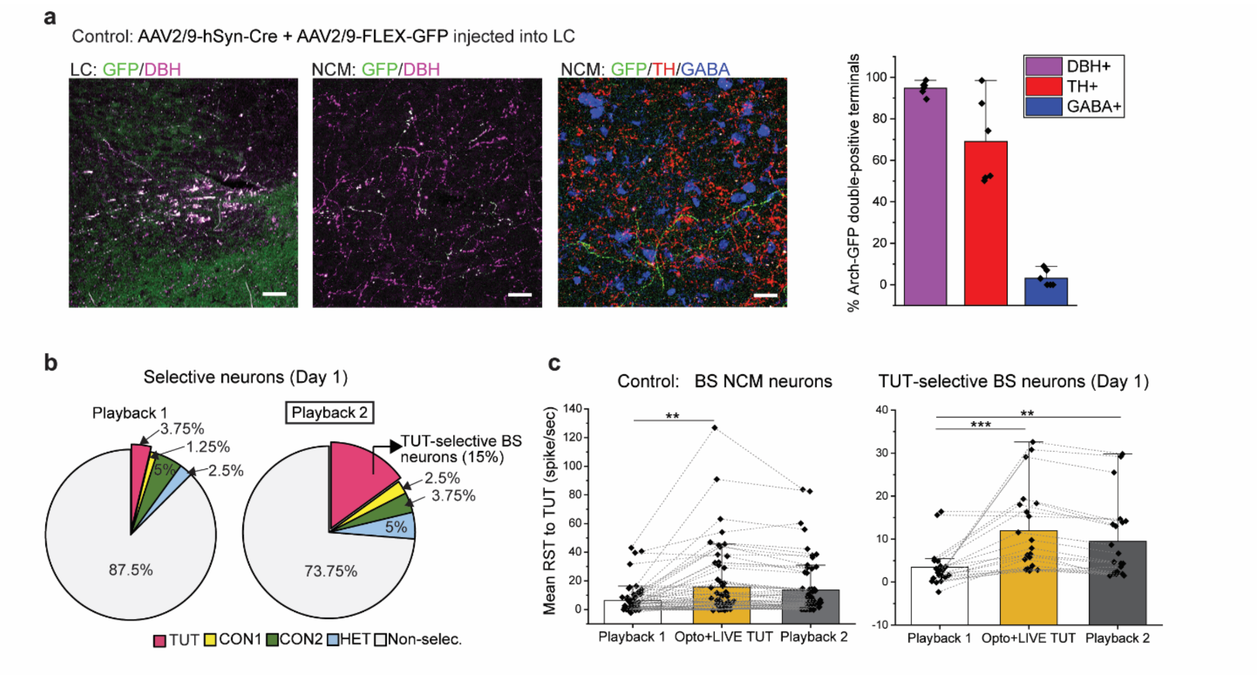

Supplementary Fig. 3: NCM neurons increase auditory responsiveness and song selectivity to tutor song playback after social interaction with a singing tutor when LC inputs to NCM are intact

a, Control group with LC neurons expressing GFP. Left: Parasagittal sections of the LC showing that LC neurons expressing GFP are dopamine beta-hydroxylase (DBH) positive (magenta). Middle: Parasagittal section in the NCM showing that LC axonal terminals expressing GFP are DBH positive (magenta; middle left) or tyrosine hydroxylase (TH) positive (red) but not GABA positive (blue; middle right) (scale bars 100 um: left, 20 um middle). Right: Proportion of GFP axon terminals that are double-positive for DBH, TH or GABA. **b**, Proportion of NCM neurons that show selectivity to one song or no selectivity (Non-selec.) before (Playback 1) and after (Playback 2) hearing the tutor singing with control laser stimulation in the NCM. **c**, Mean response strength (RST) of all BS NCM (left) or TUT selective BS neurons (right) to tutor song playback (Playback 1 and 2) or LIVE TUT with control laser stimulation in the NCM (Opto+LIVE TUT). N = 6 (**a-c**), n = 138 (**b**), n = 62 (**c** , left), n=21 (**c**, right). BS: broad-spiking neuron, TUT: tutor song, CON1: conspecific song 1, CON2: conspecific song, HET: heterospecific song, N: number of birds, n: number of neurons. Mean ± s.e.m. (**a**, **c**), **p = 0.01, ***p < 0.001, Two-sided Mann-Whitney Rank Sum Test (**c**). Source data are provided as a Source Data file.

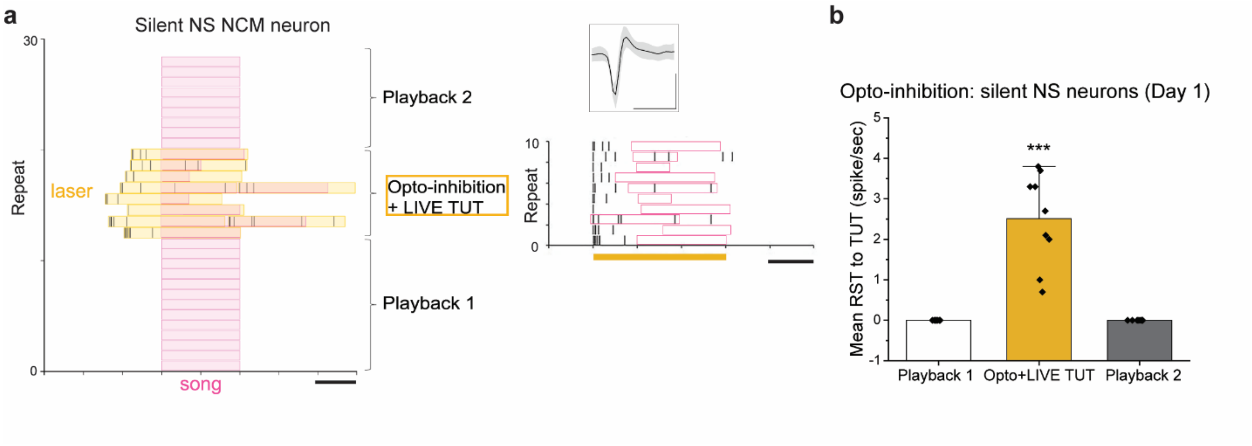

Supplementary Fig. 4: Inhibition of LC neuronal activity during social interaction with a singing tutor activates a subset of NS NCM neurons

a Raster plots of spiking activity in a silent NS NCM neuron to tutor song playback 1 and 2 or tutor singing with optogenetic inactivation of LC inputs (Opto+LIVE TUT), time-aligned to the onset of TUT playback or tutor singing (left) or laser pulses (right, yellow line indicates duration of the laser stimulation) (scale bar: 1 s). Inset: spike waveform of the same silent NS neuron (mean ± s.d., scale bars: 0.5 ms horizontal, 0.5 mV vertical). **b** Mean response strength (RST) of silent NS NCM neurons to tutor song playback (Playback 1 and 2) or Opto+LIVE TUT. N = 6, n = 9. NS: narrow-spiking neuron, N: number of birds, n: number of neurons. Mean ± s.e.m., ***$p<0.001$, Two-sided Mann-Whitney Rank Sum Test. Source data are provided as a Source Data file.

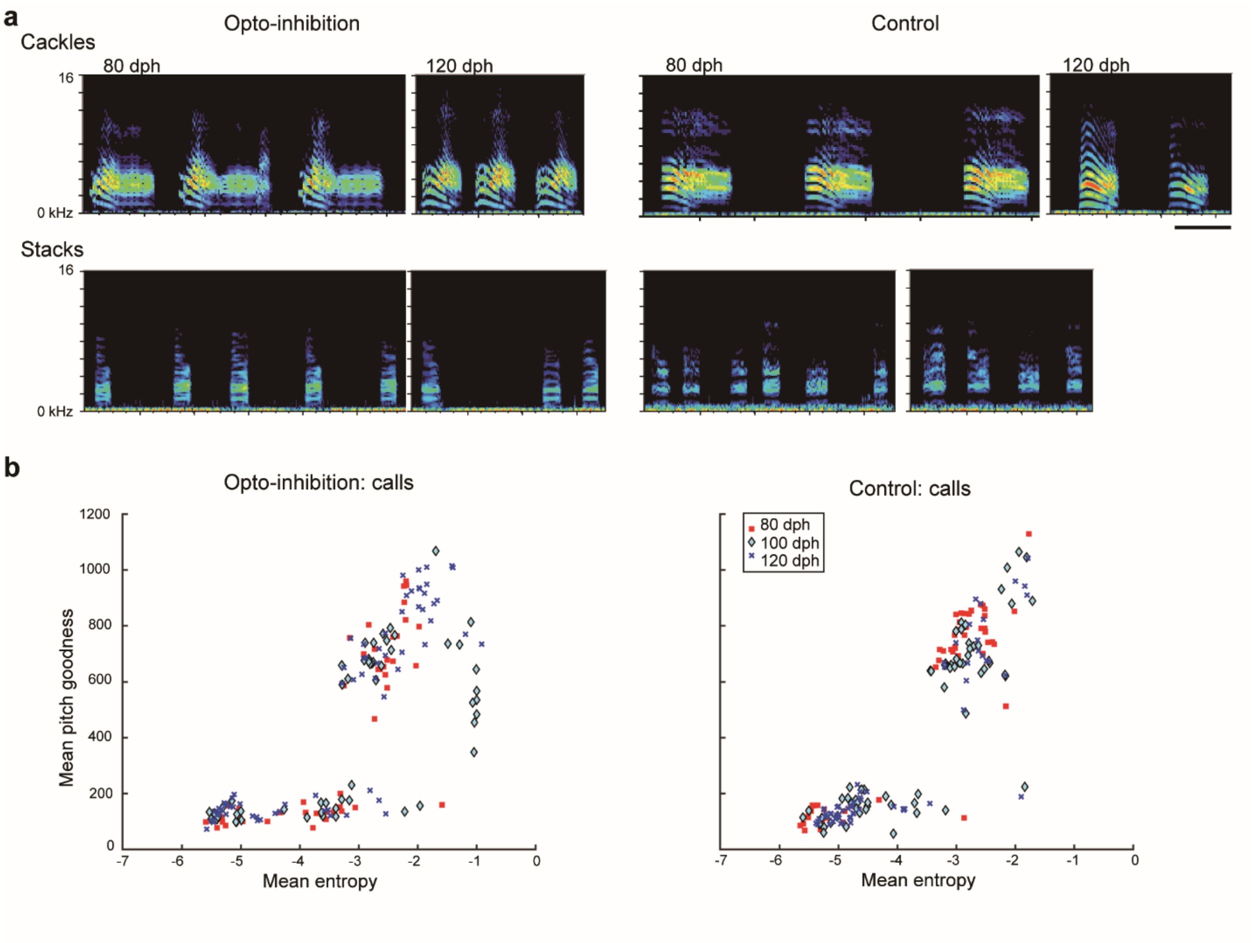

Supplementary Fig. 5: LC input inhibition in the NCM does not affect the production of calls

a, Spectrograms of cackles and stack calls from two sibling birds age 80 or 120 dph (Opto-inhibition: LC inputs were optogenetically inhibited when hearing tutor singings; Control: NCM neurons were laser stimulated when hearing tutor singing, scale bar: 0.2 s). **b**, Scatter plots of calls mean entropy and mean pitch goodness throughout call development in birds with LC opto-inhibition (left) and in control birds (right), with each dot indicating a single call. N = 6 and 6 (Opto-inhibition and Control, respectively). N: number of birds, dph: day, post hatch. Source data are provided as a Source Data file.

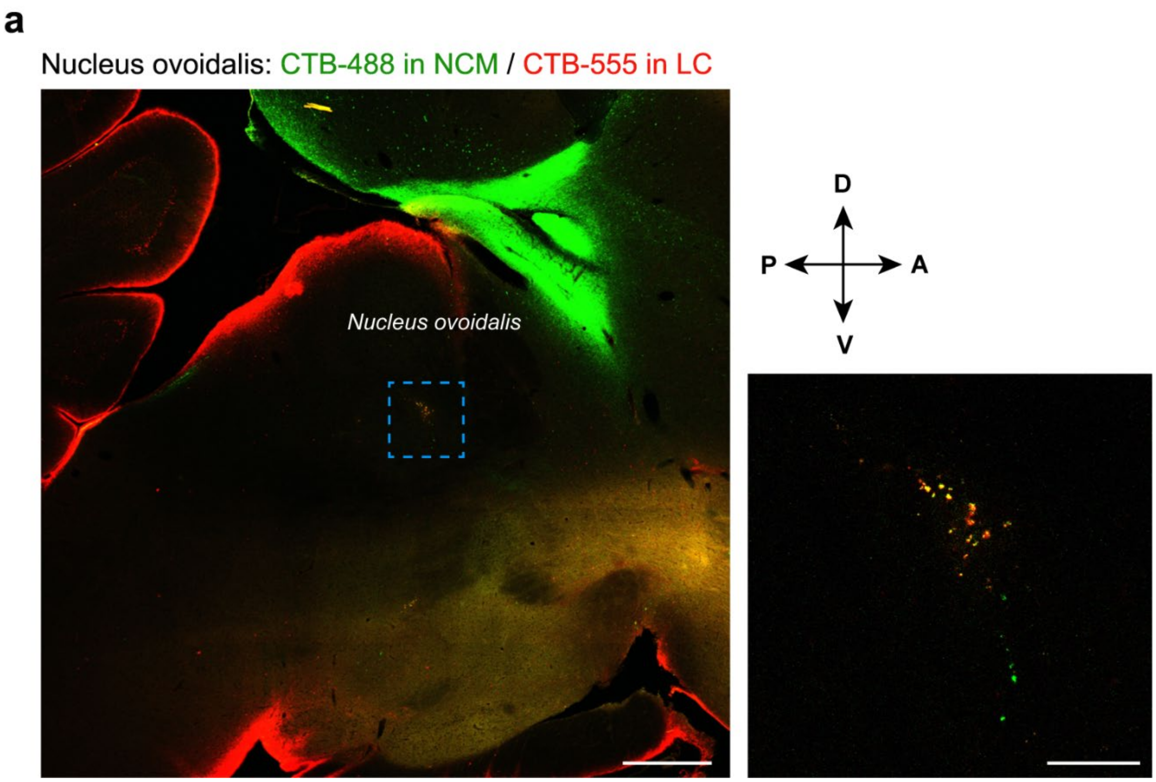

Supplementary Fig. 6: LC and NCM receive common thalamic auditory inputs

a, Parasagittal section of the thalamic region nucleus ovoidalis (white dotted circle) showing retrogradely labelled cell bodies from CTB-488 retrograde tracer injected in the NCM (green) or CTB-555 retrograde tracer injected in the LC (red). Blue dotted square (left) depicts an area magnified at 40x (right) (scale bars: 100 µm left, 20 µm right). Same results are obtained from three independent experiments using three male juvenile birds (N = 3), arrows indicate section orientation, D: dorsal, V: ventral, A: anterior, P: posterior.

Supplementary Table 1. Firing rates of neurons

		Firing rates (Hz)																
		FR (base)	FR (TUT playback 1)	FR (base)	FR (TUT LIVE)	FR (base)	FR (TUT playback 2)	FR (base)	FR (TUT playback 3)	FR (CON1 playback 1	FR (CON1 playback 2	FR (CON1 playback 3	FR (CON2 playback 1)	FR (CON2 playback 2)	FR (CON2 playback 3	FR (HET playback 1)	FR (HET playback 2)	FR (HET playback 3)
Fig. 1b, d	Neuron 1	0.60	2.57	1.89	12.11	2.02	3.37			0.73	11.61		3.15	6.18		2.40	4.14	
	Neuron 2	10.20	10.23	6.80	25.05	9.80	11.20			11.03	24.16		24.18	17.71		10.25	11.97	
	Neuron 3	0.89	0.99	1.00	6.17	0.10	2.40			1.63	1.20		3.16	4.91		1.82	8.32	
	Neuron 4	1.25	1.50	0.70	9.25	1.90	4.35			1.38	10.87		3.55	6.22		2.82	4.49	
	Neuron 5	5.58	6.11	4.80	15.31	4.40	6.46			6.66	11.57		10.95	7.68		7.31	8.59	
	Neuron 6	31.33	29.66	30.10	35.12	27.89	28.56			32.95	33.16		37.79	35.11		33.74	32.26	
	Neuron 7	4.56	5.81	5.10	41.35	7.09	18.86			6.15	13.22		7.05	14.01		5.51	10.73	
	Neuron 8	29.50	29.53	22.67	35.32	23.03	23.42			29.93	31.59		38.03	30.34		30.95	33.27	
	Neuron 9	40.96	42.41	32.09	55.26	34.50	40.53			41.15	40.31		47.04	41.01		41.11	46.14	
	Neuron 10	2.44	5.36	2.78	13.93	3.55	10.25			4.49	11.08		6.31	12.36		4.42	9.93	
	Neuron 11	26.40	31.22	29.80	53.95	32.54	42.57			28.23	40.88		28.61	42.37		29.00	44.94	
	Neuron 12	27.80	30.33	27.20	56.74	31.50	39.90			28.88	33.15		29.37	36.46		28.02	41.93	
	Neuron 13	0.98	3.95	0.88	9.79	1.17	3.80			2.72	4.67		3.96	4.81		2.60	3.85	
	Neuron 14	45.76	47.62	41.90	50.91	38.60	39.22			45.80	49.29		47.81	41.05		46.33	44.89	
	Neuron 15	1.80	7.03	3.35	30.57	3.80	14.34			2.09	10.52		6.91	8.30		2.39	9.36	
	Neuron 16	72.66	74.28	68.01	84.00	55.89	60.38			73.95	69.76		78.89	69.34		73.79	57.23	
Fig. 1c	Neuron 1	1.80	7.03	3.35	30.57	3.80	14.34											
Fig. 1e	Neuron 1	2.44	5.36	2.78	13.93	3.55	10.25			4.49	11.08		6.31	12.36		4.42	9.93	
Fig. 2a, Extended Data Fig.d	Neuron 1	0.17	0.11	1.00	3.82	1.11	6.22			0.32	3.68		0.28	2.21		0.23	1.79	
	Neuron 2	1.08	0.87	1.80	9.15	1.33	5.83			0.32	2.86		2.24	1.86		1.32	4.30	
	Neuron 3	1.40	-0.24	1.20	3.71	1.09	4.81			1.65	2.56		1.51	1.57		1.91	2.61	
	Neuron 4	0.25	0.36	0.40	12.73	0.67	5.67			1.80	1.10		1.28	-2.58		0.58	2.17	
	Neuron 5	1.17	0.83	0.80	5.58	1.00	2.36			1.33	1.22		3.57	1.36		0.56	1.42	
	Neuron 6	0.33	0.44	0.00	6.10	0.10	2.41			0.93	0.81		1.35	0.42		0.47	0.36	
	Neuron 7	3.03	7.06	3.20	21.26	2.89	9.78			2.32	3.43		2.44	4.93		3.42	2.94	
	Neuron 8	0.17	1.59	0.90	13.23	0.44	1.53			0.93	0.93		1.25	0.49		-0.37	0.71	
	Neuron 9	0.10	1.94	0.60	10.14	0.89	6.21			1.38	2.14		0.13	1.17		0.88	1.33	
	Neuron 10	0.90	1.14	1.20	8.03	1.67	7.00			0.67	2.16		2.24	3.67		0.95	6.32	
	Neuron 11	0.70	1.01	1.10	8.85	2.00	6.89			1.08	3.96		2.06	1.82		-0.27	2.25	
	Neuron 12	1.60	2.41	0.80	17.51	0.67	2.41			1.77	2.22		1.49	1.78		1.65	1.36	
	Neuron 13	0.30	1.01	0.80	3.31	0.78	1.06			-2.75	1.15		1.17	1.23		0.55	2.13	
	Neuron 14	0.90	1.95	0.50	2.62	0.89	1.09			3.92	1.15		1.08	1.03		1.07	1.44	
	Neuron 15	1.60	3.60	1.30	3.97	0.78	0.94			0.89	1.98		3.01	1.81		2.08	1.72	
	Neuron 16	1.70	1.86	1.80	2.44	1.33	1.65			3.11	3.26		2.92	2.94		2.38	2.75	
	Neuron 17	0.00	0.43	0.00	2.74	0.00	0.54			-3.14	1.80		1.36	1.30		0.31	0.91	
	Neuron 18	0.00	0.48	0.10	0.19	0.11	0.79			0.23	2.63		3.85	2.48		1.25	1.46	
	Neuron 19	2.00	2.54	1.00	4.46	1.89	2.27			-0.18	4.25		3.08	3.83		2.72	2.21	
	Neuron 20	0.30	1.32	0.60	1.44	0.44	0.65			1.40	1.86		2.37	1.81		1.33	0.91	
	Neuron 21	1.60	3.44	3.20	8.30	4.33	4.76			1.78	6.10		4.24	6.10		2.63	5.10	
	Neuron 22	2.80	3.12	3.00	3.72	2.56	3.34			1.84	3.04		4.54	3.04		3.67	3.32	
	Neuron 23	2.50	2.81	3.50	4.33	1.22	1.34			3.31	1.73		4.66	1.75		2.68	1.37	
	Neuron 24	0.70	2.60	1.20	3.00	2.78	2.91			1.19	4.32		2.23	4.22		1.15	3.02	
	Neuron 25	7.10	8.46	5.30	5.75	6.33	6.78			7.55	8.15		8.71	8.06		7.15	6.36	
	Neuron 26	3.00	4.36	5.50	5.99	3.56	3.61			3.05	6.35		5.40	5.92		4.90	3.76	
	Neuron 27	1.80	2.01	4.50	24.07	1.78	1.87			2.07	4.03		4.03	3.70		1.98	1.92	
	Neuron 28	0.00	0.21	0.60	1.45	2.33	6.83			1.20	6.25		1.68	6.25		0.45	2.58	
	Neuron 29	7.10	7.42	4.30	11.05	4.67	4.76			8.00	5.22		7.73	5.22		7.15	4.69	
	Neuron 30	0.77	1.85	1.02	2.09	2.26	1.67			3.15	7.28		3.04	7.28		2.83	2.60	
	Neuron 31	5.20	5.36	4.70	6.30	6.78	7.86			5.15	9.75		6.81	9.55		5.19	6.99	
	Neuron 32	6.90	8.80	9.50	20.21	9.00	9.03			7.17	12.04		11.47	12.11		6.97	9.08	
	Neuron 33	1.40	2.04	1.91	3.12	1.87	3.61			3.41	3.94		5.54	3.83		1.40	1.97	
	Neuron 34	0.80	0.91	0.60	2.42	1.56	6.56			2.28	4.44		3.14	4.65		1.24	2.33	
	Neuron 35	0.02	1.10	1.13	1.50	0.58	2.89			1.48	4.62		4.69	4.00		0.23	1.07	
	Neuron 36	0.50	0.61	1.40	2.34	1.67	3.02			2.65	2.75		1.87	2.45		2.27	3.43	
	Neuron 37	1.70	2.29	2.10	2.15	2.11	2.49			3.71	2.83		3.00	2.83		2.19	2.60	
	Neuron 38	11.85	21.70	8.25	19.60	13.75	22.56			32.79	35.58		16.22	20.39		23.91	24.29	
	Neuron 39	2.75	12.70	6.15	17.30	2.26	9.78			20.44	20.61		25.15	24.41		12.65	11.25	
	Neuron 40	10.42	23.20	14.42	28.40	11.56	32.33			28.43	29.92		20.66	21.49		18.68	19.50	
	Neuron 41	0.32	8.50	0.07	9.90	0.06	8.78			18.70	19.19		12.96	12.11		3.13	2.14	
	Neuron 42	8.36	17.00	5.11	16.20	6.95	21.89			28.05	28.26		31.47	20.72		18.89	14.36	
	Neuron 43	7.20	29.69	4.60	32.25	5.22	33.31			29.63	28.45		32.37	29.44		34.47	32.94	
	Neuron 44	4.07	28.70	2.01	46.90	5.23	34.33			26.11	27.71		23.86	12.67		25.66	11.52	
	Neuron 45	19.66	42.90	15.63	44.60	15.03	49.70			45.29	41.00		46.62	53.20		48.57	50.85	
	Neuron 46	1.79	24.00	2.11	37.20	2.97	39.40			16.99	18.77		19.21	20.16		21.31	18.42	
	Neuron 47	2.58	14.40	1.80	30.80	2.90	21.00			18.68	20.89		13.51	23.06		18.34	12.52	
	Neuron 48	1.84	8.30	2.68	8.60	1.79	9.10			14.15	5.30		7.27	5.59		7.18	5.83	
	Neuron 49	14.08	21.00	14.67	22.90	11.18	19.00			24.60	14.60		26.61	14.95		19.42	15.58	
	Neuron 50	12.54	20.00	6.52	25.90	12.24	20.30			23.41	19.08		21.57	16.45		28.32	18.27	
	Neuron 51	3.67	9.19	4.92	9.54	6.88	13.00			13.21	17.96		6.56	11.29		10.03	10.11	
	Neuron 52	16.71	20.70	13.01	19.70	14.06	19.60			24.32	21.30		27.42	18.84		21.81	19.06	
	Neuron 53	7.93	24.30	6.89	41.10	3.20	36.50			11.83	9.66		11.57	8.54		11.97	7.94	
	Neuron 54	7.70	39.36	5.60	49.98	6.70	43.21			11.21	9.19		11.96	12.04		12.11	11.81	
	Neuron 55	29.20	45.70	23.78	43.50	10.22	39.90			38.14	18.76		34.89	26.01		35.24	26.04	
	Neuron 56	7.00	27.38	5.30	21.46	6.30	34.84			27.67	12.38		9.98	12.66		10.23	12.53	
	Neuron 57	2.94	17.40	2.82	14.30	2.54	29.40			22.52	6.41		7.45	7.64		7.95	7.20	
Fig. 2b, g; Extended Data Fig.b, c	Neuron 1	0.17	0.11	1.00	3.82	1.11	6.22			0.32	3.68		0.28	2.21		0.23	1.79	
	Neuron 2	1.08	0.87	1.80	9.15	1.33	5.83			0.32	2.86		2.24	1.86		1.32	4.30	
	Neuron 3	1.40	-0.24	1.20	3.71	1.09	4.81			1.65	2.56		1.51	1.57		1.91	2.61	
	Neuron 4	0.25	0.36	0.40	12.73	0.67	5.67			1.80	1.10		1.28	-2.58		0.58	2.17	
	Neuron 5	1.17	0.83	0.80	5.58	1.00	2.36			1.33	1.22		3.57	1.36		0.56	1.42	
	Neuron 6	0.33	0.44	0.00	6.10	0.10	2.41			0.93	0.81		1.35	0.42		0.47	0.36	
	Neuron 7	3.03	7.06	3.20	21.26	2.89	9.78			2.32	3.43		2.44	4.93		3.42	2.94	
	Neuron 8	0.17	1.59	0.90	13.23	0.44	1.53			0.93	0.93		1.25	0.49		-0.37	0.71	
	Neuron 9	0.10	1.94	0.60	10.14	0.89	6.21			1.38	2.14		0.13	1.17		0.88	1.33	
	Neuron 10	0.90	1.14	1.20	8.03	1.67	7.00			0.67	2.16		2.24	3.67		0.95	6.32	
	Neuron 11	0.70	1.01	1.10	8.85	2.00	6.89			1.08	3.96		2.06	1.82		-0.27	2.25	
	Neuron 12	1.60	2.41	0.80	17.51	0.67	2.41			1.77	2.22		1.49	1.78		1.65	1.36	
	Neuron 1	0.30	5.90			0.11	7.01			0.73	0.90		0.95	1.06		1.66	0.59	
	Neuron 2	1.25	3.45			0.98	5.58			1.63	2.01		1.30	3.49		2.35	1.81	
	Neuron 3	1.67	14.37			1.88	14.68			1.88	2.65		2.09	3.60		2.13	2.38	
	Neuron 4	2.40	11.60			2.55	10.65			2.43	3.20		2.46	8.35		2.88	3.51	
	Neuron 5	0.00	1.80			0.10	3.10			0.49	0.42		0.11	0.71		2.00	0.35	
	Neuron 6	0.42	5.02			0.13	9.83			1.57	0.90		1.00	5.43		1.12	1.08	
	Neuron 7	2.40	8.90			2.12	10.92			2.89	2.94		4.90	3.32		1.68	3.26	
	Neuron 8	4.80	10.10			3.91	8.41			6.76	4.93		5.09	6.08		5.28	4.28	
	Neuron 9	1.90	3.87			2.50	11.30			3.15	2.90		2.17	-1.89		6.70	2.98	
	Neuron 10	1.80	2.60			1.99	10.29			3.12	2.97		2.03	4.06		2.66	2.63	
	Neuron 11	0.10	0.16			0.10	2.17			1.57	0.37		1.52	-0.96		0.32	1.02	
	Neuron 12	2.80	2.96			1.77	5.17			4.51	2.37		2.80	0.43		3.82	2.75	
	Neuron 1	1.80	13.90	1.60	11.95	1.10	11.60			0.74	2.79		2.56	4.50		0.84	1.95	
	Neuron 2	1.50	6.70	0.70	8.19	0.76	6.76			0.68	1.41		4.40	2.92		0.84	1.83	
	Neuron 3	0.70	4.80	0.00	3.45	0.20	3.50			0.61	2.54		0.80	4.30		0.82	0.86	
	Neuron 4	1.10	6.20	0.00	5.88	1.90	6.70			1.14	2.81		3.80	2.40		0.72	2.72	
	Neuron 5	3.00	12.10	2.00	10.25	2.40	7.50			0.91	3.94		4.50	2.50		0.83	3.92	
	Neuron 6	2.80	5.60	2.30	5.63	1.80	6.50			1.30	2.72		5.30	4.54		0.92	2.40	
	Neuron 7	0.12	2.82	1.60	4.45	0.58	4.68			0.91	2.58		0.12	0.67		1.10	1.29	
	Neuron 1	1.80	10.20			0.90	8.30			2.80	6.70		4.70	4.30		2.65	1.92	
	Neuron 2	0.77	4.87			0.20	5.10			1.68	1.20		1.81	-1.90		1.84	0.80	
	Neuron 3	0.70	7.80			1.00	9.60			2.81	1.20		1.15	7.50		1.66	1.31	
	Neuron 4	1.30	7.30			1.80	4.20			1.63	2.24		1.31	5.90		2.12	2.13	
	Neuron 1	1.12	4.02			0.75	8.15			1.24	0.13		2.20	1.83		1.06	0.57	
	Neuron 2	1.20	9.10			0.95	5.55			1.33	1.04		2.06	3.45		0.99	-0.86	
	Neuron 3	0.50	2.40			1.20	5.10			0.43	-3.30		1.41	2.34		-0.41	0.58	
	Neuron 4	0.80	6.50			0.20	4.90			1.25	0.29		2.83	0.21		1.02	-1.31	
	Neuron 5	0.18	1.98			0.58	4.58			0.23	1.17		0.29	1.89		-0.16	0.66	
	Neuron 1	0.40	4.22			0.40	2.73			1.37	0.83		4.50	1.64		1.01	3.52	
	Neuron 2	0.91	6.66			0.68	5.46			2.71	2.04		1.11	1.69		2.33	3.18	
	Neuron 3	0.87	5.78			1.20	6.30			2.71	2.56		1.40	1.95		1.86	2.16	
Fig. 2d, Extended Data Fig.2a	Neuron 1	0.33	0.44	0.00	6.10	0.10	2.41			0.93	0.81		1.35	0.42		0.47	0.36	

Fig. 3c	Neuron 1	1.33	7.00	0.90	4.30	1.44	3.56											
	Neuron 2	0.25	3.25	0.20	2.10	0.56	1.56											
	Neuron 3	0.15	6.48	0.19	6.09	0.40	4.84											
	Neuron 4	0.83	1.92	0.90	2.10	0.67	2.00											
	Neuron 5	0.17	1.00	0.70	0.40	0.78	0.33											
	Neuron 6	1.42	3.00	1.50	1.90	1.89	2.00											
	Neuron 7	1.33	2.25	1.70	2.40	4.22	2.89											
	Neuron 8	0.17	0.08	0.50	0.30	0.56	0.11											
	Neuron 9	0.58	2.33	0.80	2.00	1.56	1.44											
	Neuron 10	0.42	1.83	0.90	2.20	1.22	1.44											
	Neuron 11	1.00	3.08	0.70	2.80	0.89	1.89											
	Neuron 12	0.25	8.17	0.50	3.80	1.00	2.78											
	Neuron 13	0.33	0.50	0.50	0.60	0.22	0.44											
	Neuron 14	0.00	0.42	0.20	0.90	0.22	0.22											
	Neuron 15	0.67	1.83	0.40	0.90	0.78	0.89											
	Neuron 16	0.90	2.10	1.20	2.40	0.33	2.56											
	Neuron 17	1.60	4.10	1.60	3.90	1.67	4.44											
	Neuron 18	0.40	2.40	0.60	1.80	0.89	1.78											
	Neuron 19	0.30	0.00	0.70	0.00	1.00	0.00											
	Neuron 20	0.60	0.20	0.30	0.00	0.44	0.33											
	Neuron 21	0.20	0.10	0.20	0.00	0.11	0.11											
	Neuron 22	1.50	7.40	3.80	7.00	2.56	6.44											
	Neuron 23	0.20	0.10	0.30	0.00	0.89	0.11											
	Neuron 24	1.90	0.30	1.70	0.80	0.67	0.89											
	Neuron 25	0.20	1.30	0.60	1.10	0.56	1.78											
	Neuron 26	0.40	2.40	0.70	1.90	0.22	1.56											
	Neuron 27	0.90	4.40	1.70	2.40	1.78	2.00											
	Neuron 28	0.40	0.40	0.50	0.90	0.00	0.44											
	Neuron 29	0.70	4.20	1.80	4.10	2.00	3.89											
	Neuron 30	2.60	0.10	2.00	0.00	3.22	0.11											
	Neuron 31	1.20	0.50	0.80	0.30	0.78	0.11											
	Neuron 32	2.40	3.20	2.70	1.30	2.33	1.89											
	Neuron 33	0.70	0.60	0.60	2.10	0.67	2.44											
	Neuron 34	8.60	13.50	7.70	14.20	4.44	12.00											
	Neuron 35	0.10	1.80	1.00	2.60	0.56	2.89											
	Neuron 36	0.20	3.40	0.50	3.50	0.22	3.22											
	Neuron 37	0.30	1.40	0.70	1.40	0.22	0.56											
	Neuron 38	0.80	2.90	1.00	3.30	2.44	4.33											
	Neuron 39	0.70	1.90	1.90	2.80	2.56	2.56											
	Neuron 40	0.30	0.40	0.00	0.20	0.67	0.11											
	Neuron 41	1.70	2.20	1.80	4.00	2.67	2.89											
	Neuron 42	0.40	0.30	0.10	0.90	0.00	0.67											
	Neuron 43	0.40	15.60	0.50	11.00	0.67	7.11											
	Neuron 44	3.80	11.40	7.90	19.90	7.44	20.00											
	Neuron 45	1.30	11.90	1.50	11.80	2.78	13.33											
	Neuron 46	0.20	11.90	0.00	8.20	0.33	4.78											
	Neuron 47	1.60	10.70	3.90	11.50	3.22	8.89											
	Neuron 48	0.10	0.20	0.80	0.00	0.67	0.11											
	Neuron 49	2.50	4.10	2.90	6.00	3.33	5.56											
	Neuron 50	5.30	10.60	8.00	19.00	9.44	17.33											
	Neuron 51	1.30	7.20	1.20	12.00	2.00	10.89											
	Neuron 52	0.80	4.30	1.50	6.60	1.00	5.11											
	Neuron 53	1.30	6.70	0.60	3.10	0.11	2.67											
	Neuron 54	0.10	2.80	0.20	1.30	0.11	1.00											
	Neuron 55	2.70	19.00	2.60	15.80	2.78	16.11											
	Neuron 56	0.40	12.70	1.60	8.40	0.56	5.33											
	Neuron 57	0.40	3.10	1.30	2.30	0.67	1.67											
	Neuron 58	0.40	4.80	0.90	4.60	0.67	3.67											
	Neuron 59	0.80	5.30	0.90	7.90	1.33	8.22											
	Neuron 60	2.40	5.90	3.80	7.00	3.89	8.00											
	Neuron 61	2.20	8.60	5.10	7.50	3.89	8.11											
	Neuron 62	1.10	8.50	3.60	8.20	2.44	9.33											
	Neuron 63	1.20	15.30	1.20	7.00	1.56	6.56											
	Neuron 64	1.00	5.60	1.20	2.40	0.44	4.00											
	Neuron 65	2.00	23.00	2.80	19.40	2.67	17.67											
	Neuron 66	1.90	10.60	1.90	9.30	1.56	7.56											
	Neuron 67	0.20	4.40	0.60	2.10	2.56	4.11											
	Neuron 68	0.00	10.00	0.10	1.60	0.11	1.00											
	Neuron 69	0.22	0.78	0.11	1.10	0.00	0.33											
	Neuron 70	0.00	1.22	0.00	0.90	0.11	0.44											
	Neuron 71	0.20	1.40	1.10	2.70	0.44	3.00											
	Neuron 72	0.67	5.11	1.80	6.00	1.78	4.89											
	Neuron 73	0.33	2.11	0.60	3.30	0.78	2.56											
	Neuron 74	0.56	1.56	0.50	2.50	0.44	2.89											
	Neuron 75	1.30	2.20	1.20	3.00	0.78	3.56											
	Neuron 76	0.00	1.78	0.60	3.00	0.44	3.67											
	Neuron 77	0.00	0.44	0.10	1.50	0.11	0.78											
	Neuron 78	0.33	2.44	1.30	5.70	3.56	6.89											
	Neuron 79	0.56	0.78	1.30	2.10	1.78	2.00											
	Neuron 80	0.30	1.50	0.40	2.60	0.11	2.00											
	Neuron 81	0.00	2.11	0.10	3.20	0.44	2.33											
	Neuron 82	0.30	1.90	0.50	2.70	0.11	1.89											
	Neuron 83	1.44	12.00	4.50	13.60	2.22	12.22											
Fig. 3f	Neuron 1	0.15	6.48	0.19	6.09	0.40	4.84											
	Neuron 2	1.60	4.10	1.60	3.90	1.67	4.44											
	Neuron 3	8.60	13.50	7.70	14.20	4.44	12.00											
	Neuron 4	0.80	4.30	1.50	6.60	1.00	5.11											
	Neuron 5	2.70	19.00	2.60	15.80	2.78	16.11											
	Neuron 6	0.67	5.11	1.80	6.00	1.78	4.89											
	Neuron 7	0.33	2.44	1.30	5.70	3.56	6.89											
Fig. 3e	Neuron 1	0.15	6.48	0.19	6.09	0.40	4.84											
Fig. 4a, c	Neuron 1	0.25	8.17	0.50	3.80	1.00	2.78	0.80	1.60	2.20	1.93	2.19	1.50	0.32	0.78	4.70	2.73	2.99
Fig. 4b, d	Neuron 1	1.60	10.70	3.90	11.50	3.22	8.89	0.40	0.50	2.90	8.82	1.10	1.70	4.62	1.20	4.20	10.32	1.80
	Neuron 2	1.70	2.80	1.80	2.40	1.33	1.89	1.69	0.39	7.85	3.10	7.60	2.10	2.43	1.69	5.35	2.80	13.50
	Neuron 3	1.30	6.70	0.60	3.10	0.11	2.67	1.50	1.70	3.40	1.91	1.80	1.60	2.31	2.60	5.30	9.41	1.50
	Neuron 4	0.10	2.80	0.20	1.30	0.11	1.00	2.50	3.20	0.80	4.41	3.60	1.20	1.71	3.30	1.60	2.61	3.00
	Neuron 5	0.40	12.70	1.60	8.40	0.56	5.33	2.00	7.00	4.10	2.36	5.60	2.40	0.96	5.00	2.80	3.46	7.90
	Neuron 6	0.40	3.10	1.30	2.30	0.67	1.67	0.99	3.49	4.72	2.27	1.99	5.47	3.38	0.89	9.34	6.27	1.89
	Neuron 7	1.20	15.30	1.20	7.00	1.56	6.56	0.65	7.65	4.30	4.46	3.35	2.70	2.06	2.25	4.40	15.26	4.15
	Neuron 8	0.30	9.50	0.60	7.40	0.44	5.00	0.21	0.91	6.20	3.14	2.01	1.10	2.24	0.81	1.50	3.44	0.51
	Neuron 9	1.33	7.00	0.90	4.30	1.44	3.56	1.36	3.26	3.83	7.24	8.36	3.53	3.14	3.06	4.83	14.24	8.36
	Neuron 10	0.25	3.25	0.20	2.10	0.56	1.56	0.33	1.03	6.85	3.86	3.03	4.35	11.86	1.13	16.75	8.86	4.83
	Neuron 11	1.08	5.58	1.80	4.50	1.33	3.56	1.20	3.20	4.28	3.13	7.60	3.98	6.43	2.80	7.18	2.73	10.10
	Neuron 12	1.42	3.00	1.50	1.90	1.89	2.00	0.99	2.19	4.72	2.49	4.29	1.32	2.09	1.99	4.92	2.69	3.49
	Neuron 13	0.25	8.17	0.50	3.80	1.00	2.78	0.80	1.60	2.20	1.93	2.19	1.50	0.78	0.32	4.70	2.73	2.99
	Neuron 14	0.67	1.83	0.40	0.90	0.78	0.89	0.35	0.15	4.67	2.28	2.45	0.87	3.68	0.55	2.77	3.48	1.75
	Neuron 15	0.40	2.40	0.60	1.80	0.89	1.78	0.56	1.96	4.80	2.19	2.16	-0.10	2.79	1.16	5.30	1.49	3.36
	Neuron 16	0.90	4.80	1.20	4.00	1.67	2.56	1.98	2.28	1.40	8.67	2.78	2.30	1.67	3.18	1.60	14.37	4.78
	Neuron 17	0.70	9.80	3.10	7.40	2.00	4.67	1.74	2.54	0.80	4.10	4.74	2.40	3.30	2.24	4.90	4.90	7.64
	Neuron 18	0.90	4.40	1.70	2.40	1.78	2.00	0.75	1.95	3.10	5.08	6.85	1.80	3.18	0.95	2.50	14.88	5.55
	Neuron 19	0.30	1.80	0.80	0.90	0.78	1.67	0.12	0.82	0.10	3.88	0.72	2.85	6.50	2.50	3.50	7.38	1.22

Extanded Data Fig. 1d	Neuron 1	40.96	42.41	32.09	55.26	34.50	40.53										
	Neuron 2	4.56	5.81	5.10	41.35	7.09	18.86										
Extanded Data Fig. 1e	Neuron 1	26.40	31.22	29.80	53.95	32.54	42.57										
	Neuron 2	0.98	3.95	0.88	9.79	1.17	3.80										
Extanded Data Fig. 2e, f	Neuron 1	22.80	88.50	33.72	111.20	24.99	100.20			56.10	59.20		77.39	60.54		92.07	79.55
	Neuron 2	18.19	87.70	30.38	134.20	9.46	83.70			54.70	53.84		46.87	45.79		75.52	60.87
	Neuron 3	15.71	71.20	25.31	138.50	15.46	102.50			44.81	60.35		60.33	47.07		45.20	62.74
	Neuron 4	17.47	86.60	42.35	105.00	23.94	49.56			52.14	52.90		45.94	62.18		84.71	79.15
	Neuron 5	39.09	106.10	21.44	99.90	26.17	63.60			75.52	61.26		89.81	64.30		94.26	81.80
	Neuron 6	8.00	27.26	24.10	78.70	12.80	48.35			43.71	29.17		17.26	77.72		26.70	33.74
	Neuron 7	8.90	32.14	22.50	51.18	15.20	51.53			28.41	46.86		19.14	35.25		39.89	32.90
	Neuron 8	16.80	42.96	22.50	67.12	22.80	54.41			42.29	39.30		38.01	82.87		26.43	40.81
	Neuron 9	17.10	34.70	22.50	50.97	31.20	69.44			46.23	51.58		44.70	126.47		34.83	49.58
	Neuron 10	17.50	42.54	39.60	90.32	19.40	57.53			34.51	33.86		50.83	32.48		56.77	39.10
	Neuron 11	16.40	59.18	18.30	104.91	15.80	80.72			63.88	91.01		103.01	58.58		32.23	91.70
	Neuron 12	24.40	69.36	20.80	77.07	25.80	126.25			38.22	100.04		80.67	70.76		37.59	100.82
	Neuron 13	32.10	67.31	39.10	254.20	28.80	88.87			45.29	115.84		53.61	64.01		46.20	73.01
	Neuron 14	44.60	87.82	60.60	163.30	45.60	140.87			107.26	71.22		147.30	88.82		60.21	68.84
	Neuron 15	13.30	55.65	65.00	75.80	32.90	31.20			91.77	70.33		91.75	75.25		31.39	57.35
	Neuron 16	3.49	7.20	7.00	8.63	5.30	7.71			5.29	7.85		4.69	6.93		5.89	9.01
	Neuron 17	5.90	9.46	4.38	9.90	3.68	6.80			8.50	6.11		7.58	9.20		9.02	7.24
	Neuron 18	13.30	9.28	10.50	12.85	16.20	18.57			15.28	18.47		14.41	18.55		15.67	12.18
	Neuron 19	3.50	7.69	2.22	4.72	4.20	6.53			5.07	6.50		4.15	6.70		5.83	8.39
	Neuron 20	2.90	6.14	2.68	3.40	1.80	3.99			4.63	4.39		4.04	2.52		5.09	5.04
	Neuron 21	0.60	0.89	1.31	3.12	1.20	3.29			2.40	2.29		2.40	2.20		1.61	4.11
	Neuron 22	1.60	2.95	1.30	8.59	1.20	2.85			8.89	2.75		6.14	1.33		3.64	5.36
	Neuron 23	1.25	2.02	0.30	2.49	1.98	2.83			3.44	2.57		2.10	4.04		1.95	3.63
Extanded Data Fig. 3c	Neuron 1	3.90	8.90	3.20	21.20	2.63	15.63										
	Neuron 2	2.60	5.71	1.90	11.68	1.93	8.60										
	Neuron 3	3.40	8.29	6.40	24.73	3.10	16.54										
	Neuron 4	2.00	4.89	1.40	12.96	1.70	10.37										
	Neuron 5	6.10	18.10	9.20	30.09	13.10	21.99										
	Neuron 6	3.90	9.34	5.67	25.00	5.70	19.59										
	Neuron 7	0.60	2.04	1.60	7.38	0.90	5.23										
	Neuron 8	12.60	26.49	12.10	56.99	9.50	40.50										
	Neuron 9	0.11	0.33	1.00	1.44	0.89	1.11										
	Neuron 10	0.00	0.00	1.00	1.33	1.00	1.33										
	Neuron 11	0.00	0.44	0.89	1.22	0.89	0.78										
	Neuron 12	0.00	0.11	0.67	1.00	0.67	0.89										
	Neuron 13	30.70	73.81	35.80	162.69	25.10	108.88										
	Neuron 14	0.00	0.22	0.67	1.22	0.67	1.00										
	Neuron 15	11.00	14.70	8.70	15.00	8.78	13.45										
	Neuron 16	32.60	43.00	29.60	36.00	35.78	48.78										
	Neuron 17	3.90	4.40	3.60	4.50	3.44	4.33										
	Neuron 18	22.20	30.80	27.80	32.50	20.89	23.89										
	Neuron 19	0.10	0.90	0.30	0.60	0.56	0.78										
	Neuron 20	7.10	17.90	12.10	15.40	9.67	17.11										
	Neuron 21	0.10	0.10	0.00	1.20	0.11	0.22										
	Neuron 22	20.70	36.30	18.90	34.20	18.56	33.22										
	Neuron 23	12.90	21.70	13.20	24.60	11.22	25.44										
	Neuron 24	18.50	20.10	14.30	16.90	14.22	16.11										
	Neuron 25	0.90	3.40	0.30	1.00	0.44	0.44										
	Neuron 26	0.20	0.40	0.70	1.20	0.89	1.45										
	Neuron 27	19.70	51.40	22.80	61.10	18.11	55.89										
	Neuron 28	21.30	62.00	24.30	78.30	24.44	84.67										
	Neuron 29	1.70	6.10	1.60	6.90	2.89	10.67										
	Neuron 30	1.20	17.60	1.20	17.50	0.70	14.90										
	Neuron 31	0.40	5.60	0.30	2.20	0.40	0.80										
	Neuron 32	3.70	18.50	1.70	13.90	2.60	7.50										
	Neuron 33	1.40	3.60	1.00	3.90	0.70	3.10										
	Neuron 34	0.50	6.30	0.80	4.90	1.10	3.00										
	Neuron 35	1.00	13.00	0.80	11.00	0.40	13.10										
	Neuron 36	20.20	59.80	17.10	54.40	15.40	41.40										
	Neuron 37	1.40	2.30	0.44	7.00	4.70	6.20										
	Neuron 38	3.30	5.20	0.56	8.44	7.20	11.50										
	Neuron 39	0.90	1.10	0.33	4.11	2.20	4.20										
	Neuron 40	0.50	1.60	0.11	3.22	1.90	3.80										
	Neuron 41	0.50	0.60	1.80	2.10	0.00	1.89										
	Neuron 42	1.60	3.70	3.70	6.00	0.22	4.56										
	Neuron 43	1.40	0.60	2.50	2.50	0.00	3.33										
	Neuron 44	0.90	1.40	4.30	3.70	0.33	6.44										
	Neuron 45	1.20	3.50	3.20	5.20	0.22	4.44										
	Neuron 46	0.40	0.30	0.00	2.89	0.80	2.30										
	Neuron 47	1.00	1.30	2.20	1.80	0.56	5.33										
	Neuron 48	0.30	0.00	0.70	0.70	0.00	1.00										
	Neuron 49	1.50	1.10	3.40	3.20	0.56	4.78										
	Neuron 50	1.10	1.30	2.60	5.40	0.56	4.11										
	Neuron 51	1.00	2.20	2.10	2.60	0.89	3.22										
	Neuron 52	24.30	27.00	16.60	25.80	17.22	27.89										
	Neuron 53	9.30	16.50	5.20	50.90	4.49	42.99										
	Neuron 54	1.70	3.30	2.50	33.30	2.74	31.94										
	Neuron 55	3.80	8.50	4.91	51.91	4.53	46.83										
	Neuron 56	3.80	6.60	4.70	37.30	3.69	33.49										
	Neuron 57	9.90	17.20	6.51	69.71	5.52	61.42										
	Neuron 58	3.90	7.50	3.20	32.30	2.80	28.30										
	Neuron 59	6.10	13.40	4.49	48.19	3.88	40.28										
	Neuron 60	2.60	7.00	3.31	36.31	3.50	32.10										
	Neuron 61	37.50	72.80	20.10	208.50	16.36	169.46										
	Neuron 62	17.20	25.60	12.30	103.20	10.58	93.08										
Extanded Data Fig. 5a	Neuron 1	0.00	0.00	0.00	3.80	0.00	0.00										
Extanded Data Fig. 5b	Neuron 1	0.00	0.00	0.00	2.10	0.00	0.00										
	Neuron 2	0.00	0.00	0.00	3.80	0.00	0.00										
	Neuron 3	0.00	0.00	0.00	0.70	0.00	0.00										
	Neuron 4	0.00	0.00	0.00	1.00	0.00	0.00										
	Neuron 5	0.00	0.00	0.00	2.70	0.00	0.00										
	Neuron 6	0.00	0.00	0.00	3.30	0.00	0.00										
	Neuron 7	0.00	0.00	0.00	3.30	0.00	0.00										
	Neuron 8	0.00	0.00	0.00	2.00	0.00	0.00										
	Neuron 9	0.00	0.00	0.00	3.70	0.00	0.00										
