# Supplementary material for: Neural circuit for social authentication in song learning
Source: Nat Commun. 2022 Aug 16;13:4442. doi: 10.1038/s41467-022-32207-1 (PMC9381780; doi:10.1038/s41467-022-32207-1)
Supplement: Supplementary file 3 — Reporting Summary [file 41467_2022_32207_MOESM3_ESM.pdf]

nature portfolio

Corresponding author(s): Yoko Yazaki-Sugiyama

Last updated by author(s): Jun 10, 2022

Reporting Summary

Nature Portfolio wishes to improve the reproducibility of the work that we publish. This form provides structure for consistency and transparency in reporting. For further information on Nature Portfolio policies, see our Editorial Policies and the Editorial Policy Checklist.

Statistics

For all statistical analyses, confirm that the following items are present in the figure legend, table legend, main text, or Methods section.

n/a	Confirmed	
☐	☒	The exact sample size (*n*) for each experimental group/condition, given as a discrete number and unit of measurement
☐	☒	A statement on whether measurements were taken from distinct samples or whether the same sample was measured repeatedly
☐	☒	The statistical test(s) used AND whether they are one- or two-sided<br>*Only common tests should be described solely by name; describe more complex techniques in the Methods section.*
☐	☒	A description of all covariates tested
☐	☒	A description of any assumptions or corrections, such as tests of normality and adjustment for multiple comparisons
☐	☒	A full description of the statistical parameters including central tendency (e.g. means) or other basic estimates (e.g. regression coefficient) AND variation (e.g. standard deviation) or associated estimates of uncertainty (e.g. confidence intervals)
☐	☒	For null hypothesis testing, the test statistic (e.g. *F*, *t*, *r*) with confidence intervals, effect sizes, degrees of freedom and *P* value noted<br>*Give P values as exact values whenever suitable.*
☒	☐	For Bayesian analysis, information on the choice of priors and Markov chain Monte Carlo settings
☒	☐	For hierarchical and complex designs, identification of the appropriate level for tests and full reporting of outcomes
☒	☐	Estimates of effect sizes (e.g. Cohen's *d*, Pearson's *r*), indicating how they were calculated

Our web collection on statistics for biologists contains articles on many of the points above.

Software and code

Policy information about availability of computer code

Data collection	OmniPlex Software, PlexControl (Plexon)<br>Avisoft-RECORDER and Avisoft-SASLab Pro (Avisoft Bioacoustics)<br>MATLAB 2018b (MathWorks)- Custom code is deposited at: https://doi.org/10.5281/zenodo.6630127<br>LabVIEW 2016 64-bit (National Instruments)- Custom code is deposited at: https://doi.org/10.5281/zenodo.6630093
Data analysis	Offline Sorter v3 (Plexon)<br>NeuroExplorer v5<br>MATLAB 2018b (MathWorks)- Custom code is deposited at: https://doi.org/10.5281/zenodo.6630340<br>Sound Analysis Pro 2011<br>SigmaPlot 13.0<br>Origin 2019b<br>Fiji (ImageJ)

For manuscripts utilizing custom algorithms or software that are central to the research but not yet described in published literature, software must be made available to editors and reviewers. We strongly encourage code deposition in a community repository (e.g. GitHub). See the Nature Portfolio guidelines for submitting code & software for further information.

Data

Policy information about availability of data

All manuscripts must include a data availability statement. This statement should provide the following information, where applicable:

- Accession codes, unique identifiers, or web links for publicly available datasets
- A description of any restrictions on data availability
- For clinical datasets or third party data, please ensure that the statement adheres to our policy

Source data are provided with this paper. The data that support the findings of this study are included in the Supplementary Table 1. The datasets generated during this study and any additional information required to reanalyze the data reported in this paper are available from the corresponding author upon reasonable request.

Field-specific reporting

Please select the one below that is the best fit for your research. If you are not sure, read the appropriate sections before making your selection.

☒ Life sciences ☐ Behavioural & social sciences ☐ Ecological, evolutionary & environmental sciences

For a reference copy of the document with all sections, see nature.com/documents/nr-reporting-summary-flat.pdf

Life sciences study design

All studies must disclose on these points even when the disclosure is negative.

Sample size	No sample size was calculated. The minimal sample size was determined based on previous studies that have performed similar analysis (Yanagihara & Yazaki-Sugiyama, 2016).
Data exclusions	Due to the animal age and invasiveness of surgical procedures three animals died during the experiment (Fig. 1). Data collected from those animals were used only to calculate LC neuron spike waveforms in Supplementary Fig.1 panel a-c and excluded for calculation in Fig. 1b, d, as those animals were unable to finish the experiment. Data from one animal whose tutor was silent during the experiment (no singing) were also excluded from calculations in Fig.1 and Supplementary Fig.1.
Replication	Unless the animal dies during the experiment or it was exposed to a silent tutor, all attempts to replicate results were successful, all results were collected from multiple animals independently, the number of repetition corresponds to the animal (N) number in each experiment.
Randomization	In experiments including experimental groups animals were randomly assigned to experimental conditions.
Blinding	Data collection was not blinded since the same person injected viruses and performed behavioral, electro physiological and microscopy data. Collected data files, for the Control and Opto-inhibited groups, were further renamed before data analysis removing group and virus type information.

Reporting for specific materials, systems and methods

We require information from authors about some types of materials, experimental systems and methods used in many studies. Here, indicate whether each material, system or method listed is relevant to your study. If you are not sure if a list item applies to your research, read the appropriate section before selecting a response.

Materials & experimental systems

n/a	Involved in the study	
☐	☒	Antibodies
☒	☐	Eukaryotic cell lines
☒	☐	Palaeontology and archaeology
☐	☒	Animals and other organisms
☒	☐	Human research participants
☒	☐	Clinical data
☒	☐	Dual use research of concern

Methods

n/a	Involved in the study	
☒	☐	ChIP-seq
☒	☐	Flow cytometry
☒	☐	MRI-based neuroimaging

Antibodies

Antibodies used	Primary antibodies: rabbit-anti-dopamine beta-hydroxylase (Product ID: 22806, Immunostar), mouse anti-tyrosine hydroxylase antibody (Product ID: 22941, Immunostar) or rabbit-anti-GABA antibody (Product ID: A2052, Sigma-Aldrich). Secondary antibodies: goat anti-rabbit IgG antibody conjugated with Alexa 568 (Catalog # A-11036, Thermo Fisher) or goat anti-mouse antibody conjugated with Alexa 568 (Catalog # A-11031, Thermo Fisher).

Validation	Validated by manufacturer as stated on their website: for rabbit-anti-dopamine beta-hydroxylase antibody “Reacted with: Bird, Cat, Ferret, Finch, Guinea Pig, Hamster, Hatchetfish, Human, Monkey, Mouse, Pig, Quail, Rat, Sea Snail, Sparrow, Starling, Steer, Turkey, Turtle, Zebra Finch"; for mouse anti-tyrosine hydroxylase antibody “Reacted with: Alpaca (Llama), Amphibian, Anuran (Frog), Anuran (Urodele), Aplysia Californica (Sea Slug), Bee, Bird, Blowfly, Boar, Budgerigars, Canary, Cat, Chick, Chicken, Clam, Cockroach, Cod (Fish), Crab, Crayfish, Dog, Drosophila (Fly), Eel, Emu, Enegal Bichirs (Eel), Ewe, Ferret, Fish, Fly, Fox, Frog (Xenopus Laevis), Fruit Fly, Gastropod (Slug), Gastropoda, Gecko, Gerbil, Goldfish, Guinea Pig, Guppies, Hamster (Mesocricetus Auratus), Helisoma Duryi (Snail), Hen, Human, Iguana, Ilyanassa Obsoleta (Sea Snail), Lamprey, Leech, Lizard, Lobster, Locust, Mexican Axolotl (Salamander), Mollusca, Monkey (Macaca Fascicularis), Mouse, Newt, Opossum, Parrot, Periplaneta Americana (Cockroach), Phestilla Sibogae (Sea Coral), Phestilla Sibogae (Sea Slug), Phoronis Pallida (Phoronida), Pig, Pigeon, Possum, Prawn, Quail, Rabbit, Rat, Renilla Koellikeri (Sea Pansy), Rodent, Salamander, Salmon, Sea Fish, Sea Slug, Shark, Sheep, Short-Beaked Echidna, Shrew, Snail, Snake, Sparrow, Spisula (Clam), Squirrel Monkey, Starling, Stickleback, Tadpole, Trout, Turkey, Turtle, Urodele Amphibian, Water Buffalo, Worm, Yeast, Zebra Finch, Zebrafish”; for rabbit-anti-GABA antibody “ species reactivity: wide range, rat, Drosophila”.

Animals and other organisms

Policy information about studies involving animals; ARRIVE guidelines recommended for reporting animal research

Laboratory animals	Male zebra finches (Taeniopygia guttata) from 33 to 120 days post-hatch were used in this study.
Wild animals	No wild animals were used in the study.
Field-collected samples	No field collected samples were used in the study.
Ethics oversight	Animal care and experimental procedures were conducted following the experimental protocols approved by the animal care committee at Okinawa Institute of Science and Technology (OIST) Graduate University.

Note that full information on the approval of the study protocol must also be provided in the manuscript.
